# Immediate reattachment following surgical removal of tooth fragments from the lower lip: a report of two cases

**DOI:** 10.11604/pamj.2025.50.46.42131

**Published:** 2025-02-11

**Authors:** Youssef Amal, Sarah Tabbai, Hakima Chhoul

**Affiliations:** 1 Faculty of Dental Medicine of Rabat, Mohammed V University Morocco, Rabat, Morocco

**Keywords:** Case report, dental trauma, tooth fragment, lower lip, surgical retrieval

## Abstract

Dental traumas are prevalent, particularly among children and adolescents. In such dental emergencies, priority is often given to the damages caused to teeth. In contrast, the harm caused to the surrounding soft tissue may go unnoticed during the clinical examination. Dental fragments occasionally penetrate soft tissue and may cause several complications. Several complications often occur due to the penetration of tooth fragments into the tissues surrounding the oral cavity. This paper aims to present the diagnostic and therapeutic approach to dental fragments embedded in the lower lip of two patients for 2 months and 5 months, respectively. Also, it highlights the importance of the examination of soft tissues in dental trauma situations.

## Introduction

Maxillary anterior teeth are frequently injured in children as a result of traumatic falls during games. the maxillary incisors are highly vulnerable, this vulnerability is particularly due to their projection, position, and inappropriate lip envelopment [1]. When incisors are fractured, they can cause lacerations or even perforations to the soft tissues [2]. Therefore, if a fractured incisor is accompanied by lip swelling and laceration, physicians should consider the possibility of tooth fragments being displaced into the soft tissues [3]. Undetected tooth fragments may remain embedded for a long period causing infection, disfiguring fibrosis, and medicolegal complications [4]. When a fragment is encountered, bonding the fragment to the damaged tooth is the most conservative treatment option. This procedure provides better, long-lasting esthetic results by preserving the tooth's natural anatomic form, color, and texture [5]. The present article reports 2 cases of lower lip-embedded foreign bodies due to dentoalveolar trauma without patient awareness and without being diagnosed for long periods.

## Patient and observation

**Patient information:** a male patient, aged 11 years, presented to the pediatric and preventive dentistry department complaining of pain in the lower lip. No significant past medical history was reported. However, the patient´s dental history revealed a traumatic injury to the upper anterior teeth in addition to a lower lip laceration due to a fall on the floor at school 2 months previously.

**Figure 1:**
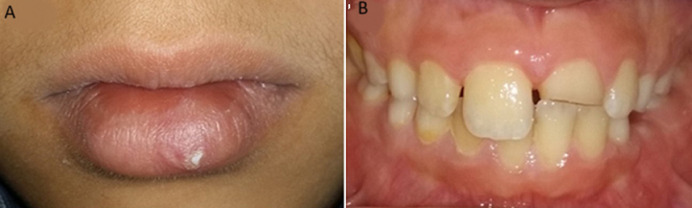
A) scaring and discoloration of the lower lip; B) the fractured central incisor

**Figure 2:**
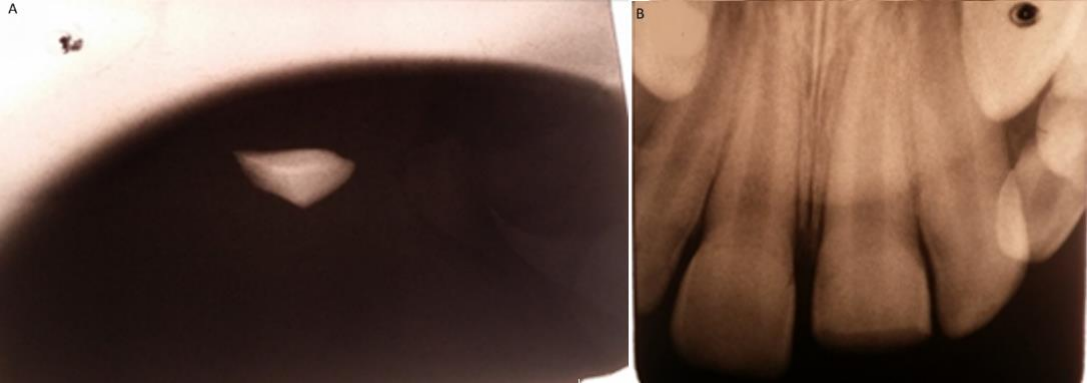
A) radiograph of the lower lip showing the radiopaque tooth fragment; B) radiograph of the central incisor

**Clinical findings:** extra-oral inspection revealed a swelling in the left paramedian of the lower lip with a transparent appearance of a foreign body (Figure 1A). On palpation, a small hard mass was felt in the lip mucosa. The intraoral examination revealed an enamel-dentin fracture of the maxillary left central incisor without pulp exposure (Figure 1B) a sensitivity test of the tooth revealed a positive reaction. A radiograph placed between the lower lip and the lower incisor teeth was taken to verify the presence of the object. The radiograph revealed a radiopaque foreign body suggestive of the coronal fragment of the fractured incisor (Figure 2A). On the periapical radiograph of the upper anterior teeth; there was evidence of an uncomplicated crown fracture, closed apex, no root fracture or pathological periapical lesion (Figure 2B).

**Diagnostic assessment:** based on the patient´s history and clinical and radiographic findings, the present case was diagnosed as a case of enamel-dentin fracture without pulp exposer with an embedded fractured tooth fragment in the lower lip following trauma.

**Figure 3:**
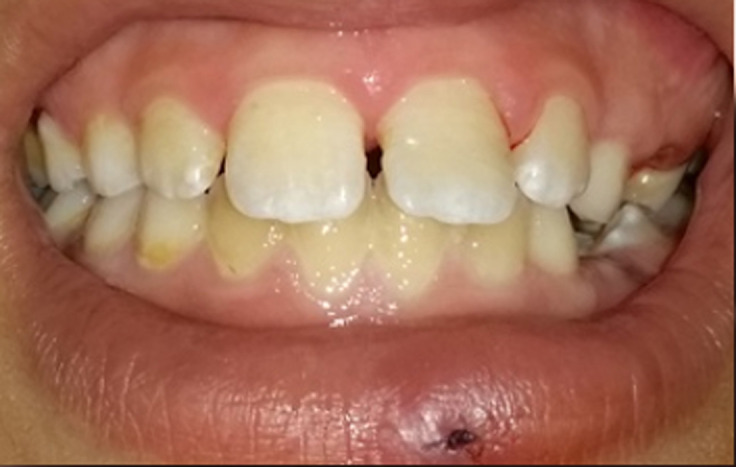
final aspect of the tooth after fragment bonding

**Therapeutic intervention:** the treatment planning strategy was carried out in two stages. In the first stage, the removal of the embedded fragment, and then, the bonding procedure and restoration were performed. In the first stage, the surgical procedure was carried out under local anesthesia. The lower lip was incised, and the tooth fragment was visualized and removed followed by suturing with 5.0 black silk suture. In order to confirm the removal of the fragment, a radiograph of soft tissues was taken in the second stage, the bonding of tooth fragments was initiated under a rubber dam. Enamel surfaces were acid-etched using a 37% ortho-phosphoric acid solution, a bonding system was applied to all treated surfaces, and the parts were approximated by hand to the original position, polymerized, finished, and polished (Figure 3).

**Follow-up and outcomes:** the patient was observed 2 weeks after the reattachment, and presented with the tooth asymptomatic, the soft tissue was healing without complaints of discomfort.

### Case 2

**Patient information:** a 15-year-old male patient reported to the department of pedodontics, complaining of pain in the lower lip that began 15 days previously. The patient´s dental history revealed traumatic injury to the upper anterior teeth in addition to a lower lip scarring that was sutured at the time by a medical practitioner.

**Figure 4:**
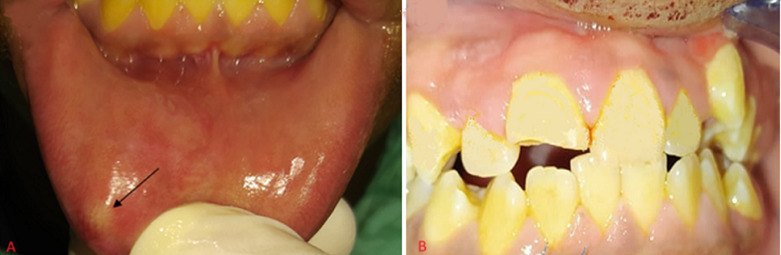
A) scaring and discoloration of the lower lip; B) the fractured central incisor

**Figure 5:**
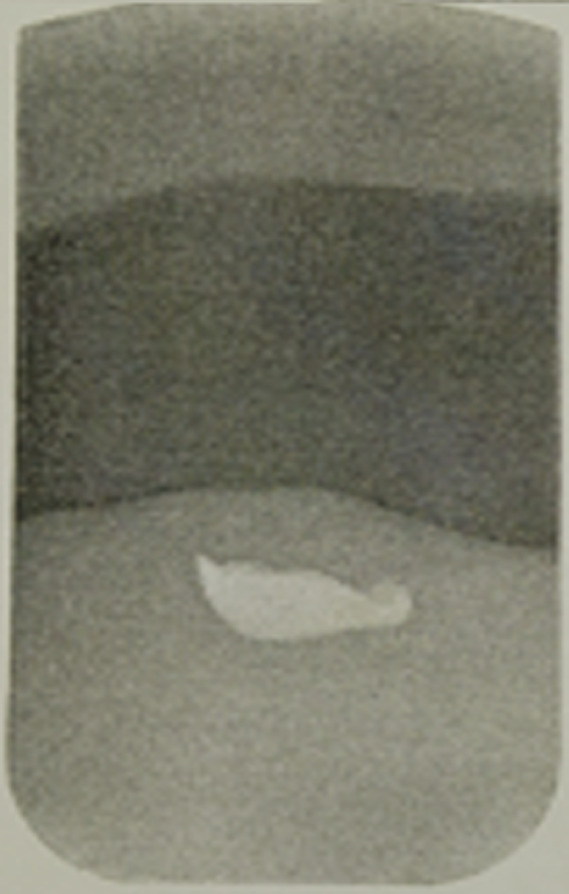
radiograph of the lower lip showing the radiopaque tooth fragment

**Clinical findings:** clinical examination showed scarring and discoloration of the left side of the external aspect of the lip without the transparent appearance of the foreign body compared to the previous case (Figure 4A). On palpation, a small hard mass was felt in the lip mucosa. Intraoral examination revealed an enamel-dentin fracture without pulp involvement in the coronary third of the maxillary left central incisor (Figure 4B). A sensitivity test of the tooth revealed a positive reaction. A radiograph of the lip was taken which confirmed the presence of a fractured tooth fragment (Figure 5).

**Diagnostic assessment:** enamel-dentin fracture without pulp exposer with embedded fractured tooth fragment in the lip.

**Therapeutic intervention:** the tooth fragment was completely removed and reattached following the same procedure as the first case.

**Follow-up and outcomes:** the patient was observed 2 weeks after the reattachment (Figure 5), and presented with the tooth asymptomatic, the soft tissue was healing without complaints of discomfort.

**Figure 6:**
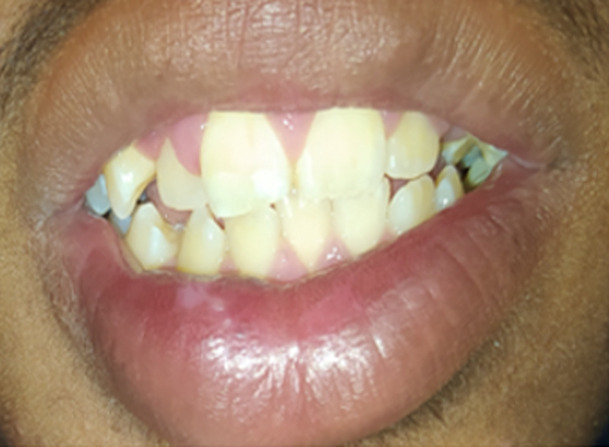
two weeks after surgical removal and bonding of the fragment

**Patient perspective:** both patients reported no pain or discomfort at follow-up and were pleased with their healing and restored teeth.

**Informed consent:** written informed consent for the case to be published was obtained from the patients and their mothers for publication of this case report, including accompanying images.

## Discussion

Dental trauma (DT) is a frequent occurrence in permanent dentition, and it can happen at any age, with a higher incidence in the first and second decades of life. Crown fractures either with or without pulp exposure represent the most frequently observed type of trauma, varying from 26.2% to 44.1% of all dental injuries [6] in children and adolescents, DT can also result in injuries to the oral soft tissue such as the lips, tongue, and gingiva. Soft tissue injuries in children were categorized as follows: lacerations accounted for 43.3%, mixed injuries (involving several types of soft tissue injuries) accounted for 30%, contusions accounted for 18.9%, and abrasions accounted for 7.8% [7]. When dealing with such cases, it´s crucial to take significant care in locating all tooth fragments that may have resulted from the accident as it is not uncommon for these fragments to become embedded in the soft tissues. The lower lip is the most frequently affected site, although examples of tooth fragments embedded in the upper lip and tongue are also reported. In this paper, cases 1 and 2 had a time gap of 2 and 5 months, respectively, between the trauma and the surgical removal of the foreign body. Ideally, diagnosing and removing a foreign body should take place in a primary care setting to avoid potential complications, extra procedures, and scar tissue formation. When a dental trauma case is presented, the diagnostic approach begins with detailed anamnesis and clinical examination. Once the broken tooth is thoroughly inspected and examined, special attention should be paid to the inspection and palpation of soft tissues that have been affected to avoid overlooking embedded tooth fragments. Clinically, tooth fragments embedded in soft tissue may be difficult to detect because of laceration and bleeding. As a result, before the wound is healed, every effort should be made to find the missing tooth structure. In such cases, a simple radiograph offers conclusive results. The radiograph is placed between the lower lip and the lower incisor teeth, with a radiographic exposure dose of 1/4^th^ of that used for standard periapical radiographs [8]. When foreign bodies remain embedded during the healing process, the risk of infection rises, resulting in the development of fibrous scar tissue. A prolonged foreign body reaction causes fibrosis, as macrophages or giant cells congregate to encapsulate the foreign body [9].

Once the embedded tooth is diagnosed after clinical and radiographic examination, a small horizontal incision is made under local anesthesia in the proximity of the hard mass, which exposes the embedded tooth fragment and allows its removal followed by bringing the edges of the wound together then sutured with simple points using 5.0 black silk for aesthetic reasons. In summary, there are multiple treatment options available for the restoration of fractured anterior teeth, including composite resin restoration and reattachment of the coronal fragment. The decision of which technique to use should be made in consultation with the patient, taking into consideration the advantages and disadvantages of each option. While composite resin restoration is highly aesthetic, it may not be able to replicate the natural tooth structure (such as translucency, opacity, opalescence, iridescence, fluorescence, and surface gloss.) as well as the reattachment of the coronal fragment, especially in cases where the fragment is not excessively restored or fragmented, which was the case in our report. Ultimately, the choice of treatment will depend on the individual patient's needs and desires, as well as the expertise and experience of the treating clinician. Very often, the loss of the reattached fragment occurs due to a second traumatic injury, not to the physiological use of the tooth. It is crucial to educate patients on the limitations and precautions required to minimize the risk of failure, including the use of mouth guards. Reattachment can also serve as a temporary solution for young patients and adolescents until they are ready for more complex prosthetic treatment. Overall, careful consideration of the individual patient's needs is necessary for successful reattachment treatment [10].

## Conclusion

In summary, beside dental and hard tissue inspection, special attention should be given to the soft tissue clinical examination even if it has been sutured and treated by another health professional during the emergency care.
